# Effect of Tartaric Acid on Hydration of a Sodium-Metasilicate-Activated Blend of Calcium Aluminate Cement and Fly Ash F

**DOI:** 10.3390/ma9060422

**Published:** 2016-05-27

**Authors:** Tatiana Pyatina, Toshifumi Sugama, Juhyuk Moon, Simon James

**Affiliations:** 1 Brookhaven National Laboratory, Upton, NY 11973-5000, USA; sugama@bnl.gov; 2 Department of Civil & Environmental Engineering, National University of Singapore, 1 Engineering Drive 2, Singapore 117576, Singapore; ceemjh@nus.edu.sg; 3 Schlumberger Riboud Product Center, 1 rue Henri Becquerel, Clamart 92140, France; james6@slb.com

**Keywords:** calcium aluminate cement, alkali activated cement, fly ash, retardation, microstructure

## Abstract

An alkali-activated blend of aluminum cement and class F fly ash is an attractive solution for geothermal wells where cement is exposed to significant thermal shocks and aggressive environments. Set-control additives enable the safe cement placement in a well but may compromise its mechanical properties. This work evaluates the effect of a tartaric-acid set retarder on phase composition, microstructure, and strength development of a sodium-metasilicate-activated calcium aluminate/fly ash class F blend after curing at 85 °C, 200 °C or 300 °C. The hardened materials were characterized with X-ray diffraction, thermogravimetric analysis, X-ray computed tomography, and combined scanning electron microscopy/energy-dispersive X-ray spectroscopy and tested for mechanical strength. With increasing temperature, a higher number of phase transitions in non-retarded specimens was found as a result of fast cement hydration. The differences in the phase compositions were also attributed to tartaric acid interactions with metal ions released by the blend in retarded samples. The retarded samples showed higher total porosity but reduced percentage of large pores (above 500 µm) and greater compressive strength after 300 °C curing. Mechanical properties of the set cements were not compromised by the retarder.

## 1. Introduction

Due to their resistance to high temperatures and aggressive acidic environments, high-performance ceramic materials are of great interest for use in constructing high-temperature geothermal wells. *In situ* synthesis of such materials from fly ash especially is attractive because of the latter’s availability, good workability, and compatibility with various cementitious materials. The use of fly ash in cementitious materials also contributes to abating the environmental impact of Portland cement, such as the release of CO_2_ and NO*_x_* during its manufacture. The low reactivity of fly ash under ambient conditions is not a limiting factor for its downhole applications in high-temperature geothermal wells.

Arguably, in geothermal wells, cement experiences the most severe conditions possible [1]. In addition to the chemically aggressive environment, the cement may suffer a significant thermal shock when wells constructed under relatively mild temperatures of about 80 to 110 °C are moved into production, and the temperature may rise by several hundred degrees. Differences in temperature between the injection- and production-heat-carrier fluids may further impose a constant thermal-shock on the cement.

Thermal-shock-resistant cement (TSRC), consisting of two cement-forming components, calcium aluminate cement (CAC) and Class F fly ash, and one alkaline activator (sodium metasilicate), was formulated to resolve this issue [2]. The TSRC autoclaved at 200 °C withstood five cycles of 500 °C heating-water quenching, sustaining its compressive strength.The formation of a densified structure made of three crystalline phases, γ-Al_2_O_3_, calcite, and carbonated sodalite, was responsible for such resistance to thermal shock. 

The alkaline activation of fly ash F (FAF) has been the subject of various studies [3,4,5,6,7,8,9,10]. Cements prepared with fly ash were shown to perform better under high temperature and in aggressive solutions than did traditional Portland or sulfate-resistant cements [11,12]. Under the most studied conditions, the main reaction product of alkaline activation of fly ash F was alkaline aluminosilicate gel, which is a zeolite precursor [13,14,15].

The formation of zeolites involves crystallization of the gel; conditions required for this are not usually reached during the activation of alkali fly ash at temperatures below 90 °C [16] due to the fast reaction rates of the dissolution and condensation processes and a very slow rate of crystallization after the material hardens [15].

CAC serves as a source of reactive aluminum during the alkaline activation of aluminosilicate materials to form binding (C,N)–A–S–H gel that ensures the early development of strength [17,18,19,20]. Aluminosilicates and CAC affect each other’s hydration in an alkaline medium. A study of the alkaline activation of metakaolin as the aluminosilicate material in the presence of CAC as a source of reactive aluminum at short hydration times at 85 °C revealed that calcium aluminate does not undergo its normal hydration with the formation of calcium aluminate hydrates and gibbsite. Instead, Al and Ca from CAC are incorporated into the aluminosilicate gel formed during the hydration of metakaolin [4].

The microstructure, nature, and chemical composition of the reaction products of both CAC and FAF depend on the alkaline activator [21,22]. The sodium-silicate activation of fly ash increases both the condensation degree of the alkaline aluminosilicate gel and its mechanical strength by promoting the gel formation during hydration of FAF [22]. On the other hand, sodium silicate and reactive silica, in particular, slow down the hydration kinetics of CAC [23,24,25,26,27,28].

For the subterranean use of cementitious materials, their hardening must be postponed or retarded to assure the necessary pumping time for placing the material in the well. The CAC and FAF set delay provided by the sodium-silicate activator is insufficient for the commonly required 4 to 6 h of pumping time. The effectiveness of various organic set-retarding additives was reported for applications with calcium aluminate cement and its components [29,30,31,32,33]. Tartaric acid was identified as the most effective retarder for TSRC at 85 °C [23].

Adding a retarder to TSRC undoubtedly will change the hydration kinetics and may affect the hydration products. The effect of retarding additives on the early-age morphology and mineralogy of cement hydration products was previously reported [34,35,36].

For the alkaline activation of fly ash, the slower kinetics of product formation and crystallization may result in a stronger material. The excellent bonding and adherent properties of alkali-activated fly ash have been attributed to the formation of alkaline aluminosilicate gel with an amorphous-nanocrystalline structure compared with the well-defined crystal structure of zeolites [14]. A more polymerized gel with lower content of zeolites contributed to creating a material with higher mechanical strength in this study. On the other hand, delayed crystallization of CAC in the presence of soluble silica may favor the formation of calcium aluminosilicate hydrate (C,N–A–S–H) gel that may crystallize into zeolitic phases [19]. 

The slower hydration kinetics of a retarded cement slurry may entail a different set temperature that will strongly affect the final material’s mineralogy [37]. With few exceptions, geothermal wells are not cemented at high temperatures. The fluids circulated during drilling cool the formation. The maximum circulating temperatures during cementing seldom exceed 116 °C [1] with the average being approximately 85 °C. After the placement of the cementitious material, the fluid circulation stops, and the temperatures increases to the static conditions of 150 to 300 °C. The following temperature conditions of cement hydration may be expected in geothermal wells: mixing at ambient temperatures, hardening at moderate temperatures of approximately 85 °C, and long-term exposure to high temperatures between 150 and 300 °C. Ideally, a fast set takes place after the slurry placement with the minimal phase transitions in the set slurry that could compromise mechanical properties of the set cement. For large variations in curing temperatures a retarder imposing significant effect on the interactions in a slurry and cement hydration kinetics may strongly affect set cement cement composition and properties.

The present paper focuses on the effect of a known CAC set retarder, tartaric acid, on mechanical properties, the mineralogy, and the morphology of a CAC-fly ash blend activated with sodium metasilicate after curing at elevated temperatures of 85, 200, and 300 °C and under the respective pressures of 0.1, 6.89 and 8.27 MPa. Although elevated pressure may affect the kinetics of cement hydration and set cement properties, the temperature effect is generally much more important [38,39,40]. The current study did not evaluate the effect of pressure on properties of the specimens. 

## 2. Materials and Methods

### 2.1. Materials and Samples Preparation

FAF was obtained from Boral Material Technologies, Inc. (San Antonio, TX, USA). A sodium metasilicate granular powder under the trade name “Metso Beads 2048”, supplied by the PQ Corporation (Malvern, PA, USA), was used as the alkali activator of FAF. Secar #80, supplied by Kerneos Inc., (Chesapeake, VA, USA) was used as the calcium-aluminate cement. The X-ray powder diffraction (XRD, PANanalytical, Almelo, The Netherlands) data showed that the crystalline compounds of FAF consisted mainly of quartz (SiO_2_), mullite (3Al_2_O_3_·2SiO_2_), and hematite (Fe_2_O_3_), and CAC included three crystalline phases, *i.e.*, corundum (α-Al_2_O_3_), calcium monoaluminate (CaO·Al_2_O_3_, CA), and calcium dialuminate (CaO·2Al_2_O_3_, CA_2_). Table 1 shows the elemental composition of the TSRC components (major oxides composition measured by EDX (Shimadzu, Kyoto, Japan) and normalized to 100%).

The blend cement consisted of 60% CAC and 40% FAF (mass fractions). The sodium metasilicate was dry-blended with this mixture at 6.0% mass fraction of the cement blend to form the TSRC blend. The water-to-solid ratio was 0.52. D-(-)-tartaric acid (TA) was supplied by Sigma-Aldrich. The retarder was dry-blended with the TSRC at mass fraction of 1- or 1.8-% of the TSRC blend. The dry blends were hand-mixed with water.

To prepare samples for measurement of compressive strength and determination of water-fillable porosity, the slurries were cast into cylindrical molds (25-mm diameter and about 40-mm length). The molds were placed into an autoclave partially filled with water (20% of volume) at 85 °C for a 3-d curing, and then the temperature was increased to the final 200 or 300 °C for the desired duration of curing. To understand whether hydrates formed at 85 °C persist in samples cured at high temperatures some samples remained at 85 °C for 22 days before analyses of their compositions. The sample with 1.8% TA could not be prepared at 85 °C because of a very slow cement set at this retarder concentration and pore slurry stability. Table 2 summarizes the curing conditions. 

### 2.2. Testing Methods

**Figure 1:**
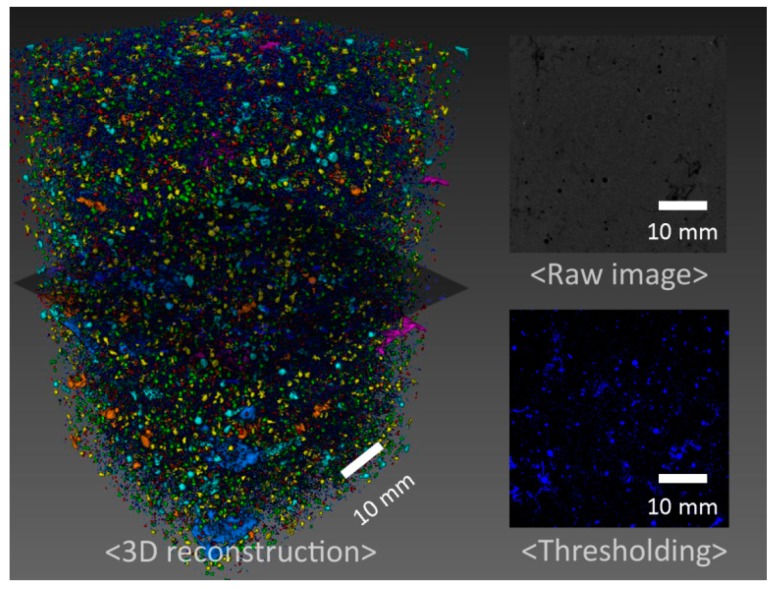
3D pore-structure reconstruction of 1.8% TA 200 °C sample from image-analysis technique.

**Figure 2:**
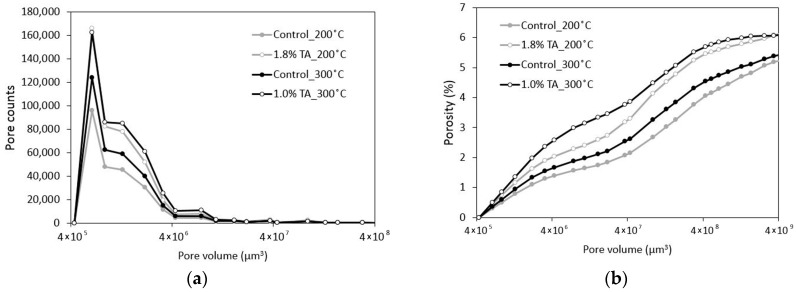
(**a**) Calculated pore counts; and (**b**) related cumulative volume (porosity).

X-ray computed tomography (CT) experiment was performed on 4 selected samples (control 200 °C, 1.8% TA 200 °C, control 300 °C, and 1% TA 300 °C) using a SkyScan 1173 CT scanner (Bruker, Kontich, Belgium). No additional treatment was required for the non-destructive CT experiment. After the target curing, the samples had been kept for 180 days at ambient conditions until CT testing. Voltage of 130 kV and current of 60 μA were selected for measurement. In addition, each scan took around 2 h with the exposing time of 500 ms and sample rotational step of 0.2 degree. Obtained 2D images have a size of 2240 × 2240 pixel^2^ with a resolution of 35.15 µm^3^/voxel. Figure 1 shows 3D pore-structure reconstruction of 1.8% TA 200 °C sample. Similar procedure was applied previously [41]. By visual inspection, a threshold value of 30/256 was reasonably selected and applied to all CT samples for pore segmentation [42]. Next, pore counts and related cumulative volume (porosity) were calculated as shown in Figure 2. Measured distribution of pore volume ranges from 4 × 10^5^ to 4 × 10^9^ μm^3^. The measured four samples show similar distribution trends regarding pore counts and partial porosity. Correlation of this data with mechanical strength is discussed in the following Section 3.

The specimens were tested for their strength right after the end of the target curing. After the strength tests, they were ground into very fine powder and dried at 90 °C for 24 h prior to thermogravimetric testing and X-ray diffractometric characterization. The former analysis was done on approximately 10 mg of sample heated at a rate of 20 °C/min in N_2_, using TGA-Q50 from TA Instruments. The samples were examined using a Philips XRG 300 X-ray diffractometer with a 40 kV, 40 mA copper anode X-ray tube. The results were analyzed using the PDF-4/Minerals 2013 database of the International Center for Diffraction Data. The morphologies of the cements were studied on typical spots of freshly fractured samples with a JEOL 7600F scanning electron microscope equipped with an EDX Oxford Link microanalysis system. The specimens were dried at 90 °C for 24 h after the curing, placed on metallic holders and coated with chromium to eliminate samples’ charging. The morphologies studies were performed within a week of the target curing.

## 3. Results

### 3.1. Mechanical Properties and Tomography Study

Table 3 summarizes short-term mechanical properties of the control (nonretarded) and TA-retarded blends after 3-d curing at 85 °C and 1-d curing at 200 or 300 °C. In practice the fast development of the short-term compressive strength allows resuming well-construction and is a desirable feature. According to the data, there was a decline in compressive strength as the curing temperature increased from 200 to 300 °C. The loss in strength was less important for the retarded specimens—54% for the control, 35% for 1% TA, and 10% for 1.8% TA. The retarded specimens also were less brittle after curing at 300 °C; the compressive-toughness of the control was 0.08 N-mm/mm^3^ 0.31 N-mm/mm^3^ for the 1% TA-retarded cement and 0.35 N-mm/mm^3^ for the 1.8% TA-retarded cement.

**Figure 3:**
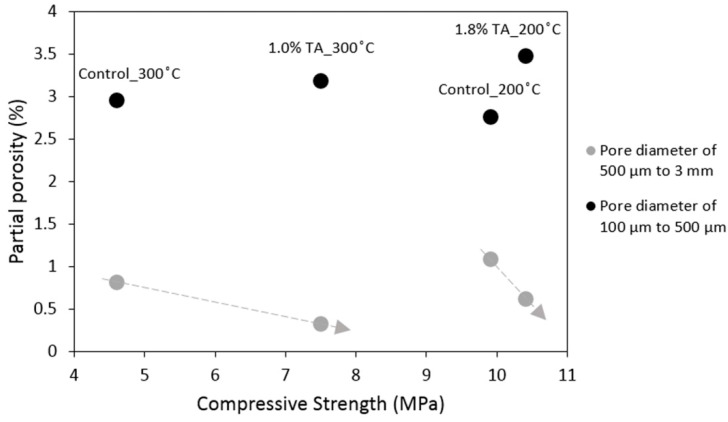
Compressive strength *vs.* calculated partial porosity for pores of small (100 µm to 500 µm) and large (500 µm to 3 mm) diameters in control and retarded TSRC specimens.

In CT measured samples, the addition of the retarder increased the porosity. This could be a result of slower fly ash F hydration and precipitation of the hydration products that fill the pores. Interestingly, the increase of total porosity (Table 3) was not accopamied by a drop in compressive strength. However, computed partial porosity separated by pore diameter of 500 microns can explain changes in compressive strength. Figure 3 shows that in each curing condition, porosity from large pores (diameter of 500 microns to 3 mm) was inversely proportional to the compressive strength (gray lines). It also suggests that the partial porosity from large pores can play a more significant role in the strength although smaller pores (diameter of 100 to 500 microns) had a larger volumetric contribution to the total porosity. Overall, it can be proposed that the use of the retarder increased the total porosity but in all cases reduced large pores, which seems to contribute to the strength gain.

### 3.2. XRD Study

**Figure 4:**
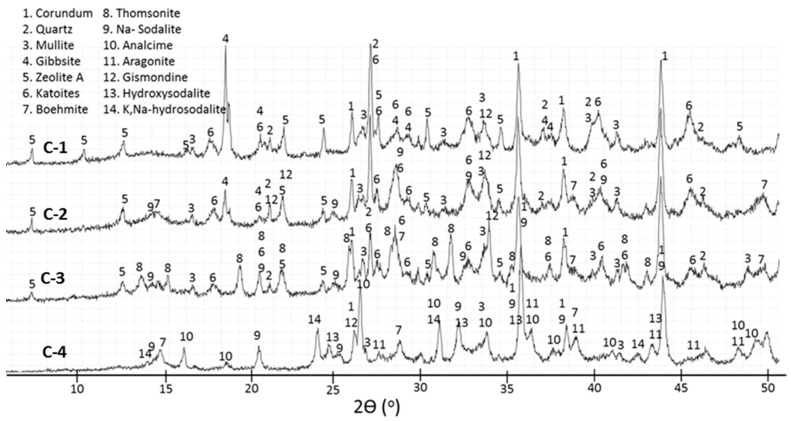
Diffractograms for control TSRC specimens.

**Figure 5:**
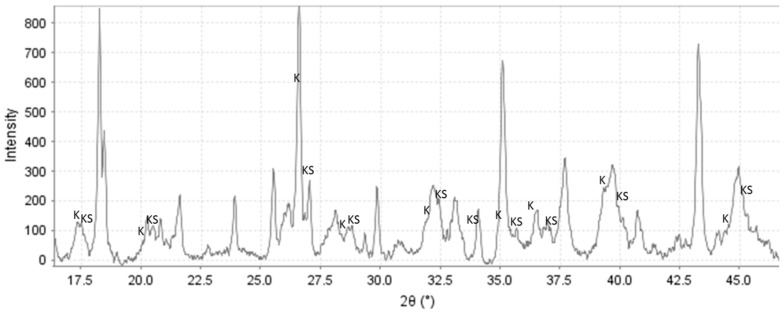
Diffractograms for C-1 specimen showing peaks of katoite (K; ICDD-00-024-0217) and katoite silician (KS; ICDD-00-038-0368).

**Figure 6:**
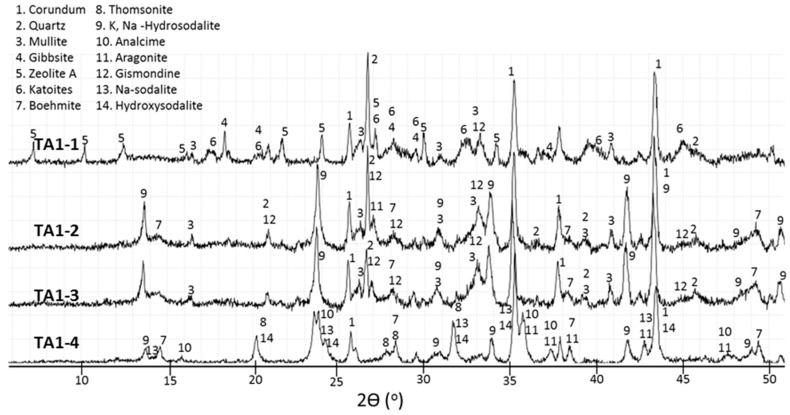
Diffractograms for TSRC specimen with 1% tartaric acid.

**Figure 7:**
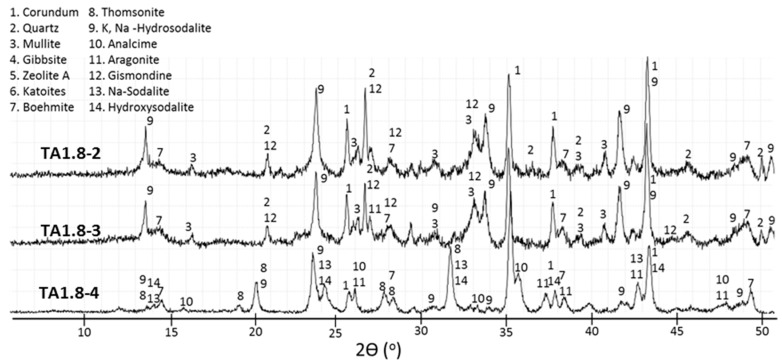
Diffractograms for TSRC specimen with 1.8% tartaric acid.

The XRD findings for 0-, 1.0-, and 1.8% TA are shown in Figure 4, Figure 5, Figure 6 and Figure 7. Corundum from CAC (ICDD: 00-005-0712) and, mullite (ICDD: 00-015-0776) and quartz (ICDD: 00-005-0490) from FAF were present on all the XRD patterns at 85 °C and 200 °C. However, at 300 °C the XRD patterns were free of quartz and mullite lines for all the specimens, strongly suggesting that these crystalline compounds in FAF reacted at this temperature. The crystalline products formed from cement hydration and fly ash activation included calcium aluminate hydrate phases (katoite Ca_3_Al_2_(OH)_12_ (ICDD: 00-024-0217) and katoite silician Ca_3_Al_2_(SiO_4_)(OH)_8_ (ICDD: 00-038-0368)), aluminum hydroxide phases (gibbsite Al(OH)_3_ (ICDD: 00-007-0324) and boehmite γAlO(OH) (ICDD: 04-013-2972)), and calcium-, sodium-containing zeolite phases (zeolite A Na_4_Ca_4_ Al_12_Si_12_O_48_(H_2_O) (ICDD: 00-038-0241), gismondine CaAl_2_Si_2_O_8_·4H_2_O (ICDD: 00-020-0452), hydroxysodalite Na_4_Al_3_Si_3_O_12_(OH) (ICDD: 00-011-0401), sodium-sodalite Na_6_(AlSiO_4_)_6_·4H_2_O (ICDD: 00-042-0216), K, Na-hydrosodalite, (Na_0.2_K_0.8_)_6_(AlSiO4)_6_·(H_2_O)_8_ (ICDD: 01-073-5304), thomsonites NaCa_2_Al_5_Si_4_O_20_·6H_2_O (ICDD: 00-046-1448 and 01-079-6019), and analcimes Na_2_Al_2_Si_4_O_12_·2H_2_O (ICDD: 00-002-0417, 00-041-1478, 00-019-1180)). Some peaks in 300 °C-cured specimens were attributed to aragonite (CaCO_3_ (ICDD: 01-080-2786)).

The crystalline phases that can be related to the Ca released by CAC were katoites, gismondine, thomsonite, and zeolite A, as observed by other authors [19], along with aluminum hydrates formed with aluminum from CAC. For the control specimen, gibbsite dominated the pattern after 22 days at 85 °C (Figure 4, C-1). Although the gibbsite peak’s intensity declined after 3 days at 85 °C and 1 day at 200 °C, it still was clearly detectable, along with the smaller peaks of high-temperature-stable boehmite. Only the boehmite peaks remained after 7-d-curing at 200 °C (Figure 4, C-3), and boehmite had become one of the major products in the 300 °C-cured specimen (Figure 4, C-4).

The presence of split katoite peaks indicates inclusion of (SiO_4_) in the structure with the formation of katoite silician (ICDD-00-038-0368) in specimens cured at 85 °C and 200 °C (Figure 5), in agreement with earlier studies [19,43]. The intensities of the katoite peaks decreased after extended autoclaving at 200 °C. Thus, the intensity of the katoite peak at 2θ 45.01°, where there was no contribution of other phases, normalized to the intensity of the corundum peak at 2θ 37.71°, was about 65% lower after 7 days than after 1 day at 200 °C. The katoites vanished after curing at 300 °C.

Zeolite A was one of the major crystalline products in the control specimen cured at 85 °C, as was also observed by others in studies of the alkaline activation of FAF [4], and after the hydration of CAC in the presence of sodium silicate [19]. Zeolite A was still present after curing at 200 °C, but the intensities of its peaks (Figure 4, C-2 and C-3) clearly decayed, and the pattern of the 300 °C-cured specimen was free of zeolite A peaks (Figure 4, C-4). Thomsonite replaced zeolite A, and it became one of the major products after 7 days at 200 °C (Figure 4, C-3). Analcime, in turn, replaced thomsonite at 300 °C (Figure 4, C-4). Thus, the following phase transitions occurred between 85 and 300 °C:

Zeolite A (Na_4_Ca_4_ Al_12_Si_12_O_48_(H_2_O) → thomsonite (NaCa_2_Al_5_Si_4_O_20_·6H_2_O) → analcime (Na_2_Al_2_Si_4_O_12_·2H_2_O).

Additionally, although gismondine was a minor phase, its disappearance at 300 °C may be a result of the gismondine (CaAl_2_Si_2_O_8_·4H_2_O) → analcime (Na_2_Al_2_Si_4_O_12_·2H_2_O) transformation. The peaks of hydroxysodalites were found in specimens cured both at 200 and 300 °C, but not at 85 °C. The crystalline hydroxysodalite was a minor product compared with thomsonite in the 200 °C-cured specimen (Figure 4, C-3). At 300 °C, the latter had disappeared whereas peaks of hydroxysodalite were still present. Sodium sodalite (ICDD-00-042-0215) and analcime matched most of the peaks of this XRD pattern (Figure 4, C-4).

Gismondine may also be present in samples cured at 85 and 200 °C. The *d*-spacings of gismondine overlap significantly with those of katoite, but only mullite from FAF and gismondine contribute to the peak at 2θ of 33.2°. This peak of mullite is not the major one, and would be expected to be smaller than the main peak at 26.16°; however, this is not the case, suggesting another contributor to the peak’s intensity. In addition, the intensity of the peak at 33.2° increases after curing at 200 °C, whereas there is no reason to expect an increased intensity of the mullite peaks. These arguments support the presence of gismondine in all of the specimens, except those cured at 300 °C at which temperature its peaks cannot be confirmed because they are absent or strongly overlap with the analcime peaks. Since the thermal decomposition of gismondine takes place between 200 and 300 °C, its presence in 300 °C-cured samples is unlikely [44].

Thomsonite contains both sodium and calcium, and gismondine is a calcium-based zeolite. The XRD patterns do not allow distinguishing between zeolite A (ICDD-00-038-0241) and zeolite A, Ca (ICDD-04-010-2002), so the presence of a calcium-containing zeolite A cannot be excluded. If fly ash and the sodium silicate activator are likely sources of sodium and silicon, calcium is mostly supplied by CAC since the content of calcium in fly ash is very low. Aluminum released during the CAC hydration is responsible for the formation of gibbsite (Figure 4, C-1 and C-2) and boehmite (Figure 4, C-3 and C-4), while calcium participates in forming the zeolite phases. If this is the case, then the zeolite phases in the hydrated cement are produced by ions from both CAC and sodium-silicate-activated FAF. Crystallization of calcium-containing zeolites from a gel formed by the alkaline activation of FAF and the uptake of Al and Ca ions from CAC was shown for alkaline-activated blends of metakaolin and calcium-aluminate cement [18].

Thus, the control cement sets at 85 °C with the formation of crystalline products that persist into the high-temperature domains where these phases are not stable, so resulting in slow phase transitions. Several phases formed in the control sample after 1-day curing as major phases become minor after 7 days at 200 °C. For instance, boehmite replaces gibbsite, and the intensities of the zeolite A and katoite peaks decrease while those of hydroxysodalite and gismondine increase.

The XRD pattern of TSRC retarded with 1% TA and cured for 22 days at 85 °C contains the main crystalline products similar to those in the control specimens (Figure 6). However, the peaks are generally smaller, especially that of gibbsite. The intensity of the gibbsite peak at 2θ 18.34 normalized to corundum peak at 2θ 37.71 is about 90% lower than that of the control sample. In addition, there is a halo between 2θ of 48° and 50° on the pattern of the 1% TA sample that is absent in the pattern of the control. This halo may be from a poorly crystallized precursor of boehmite.

Contrary to the control specimen, zeolite A, katoite, and gibbsite do not survive the curing at 200 °C with 1% tartaric acid; this may be a result of their reduced crystallinity in the retarded specimen.

On the other hand, the newly formed peaks of K,Na-hydrosodalites appear to be much stronger in retarded specimens after curing at 200 °C (Figure 6 and Figure 7). Thomsonite does not crystallize in the presence of the retarder, even after autoclaving the specimen for a week at 200 °C, but it is found in the pattern of 300 °C-cured cement. The analcime peaks are much smaller for the 300 °C-cured sample with 1% TA, suggesting a lower amount or/and lower crystallinity of this phase in the presence of the retarder.

For both TA concentrations, the XRD patterns are very similar after curing at 200 and 300 °C (Figure 6 and Figure 7). The peaks of Na-sodalites and analcime are smaller in the 300 °C-cured sample at higher concentration of the retarder.

Unlike for the control sample there are fewer crystalline phases transitions for the retarded specimens with increasing temperature and none takes place during longer curing at 200 °C. It is clear that the delayed set of the retarded cement results in formation of products stable at the final curing temperature; specifically, boehmite, gismondine, and K,Na-hydrosodalite form both in samples cured at short, 1-d curing periods and longer, 7-d ones at 200 °C. Furthermore, the use of retarder seems to promote crystallization of certain phases while others are inhibited. Accordingly, the crystallization of thomsonite seems to be repressed by the retarder while Na-sodalite gives peaks of higher intensity than those in the control samples.

### 3.3. TGA Study

**Figure 8:**
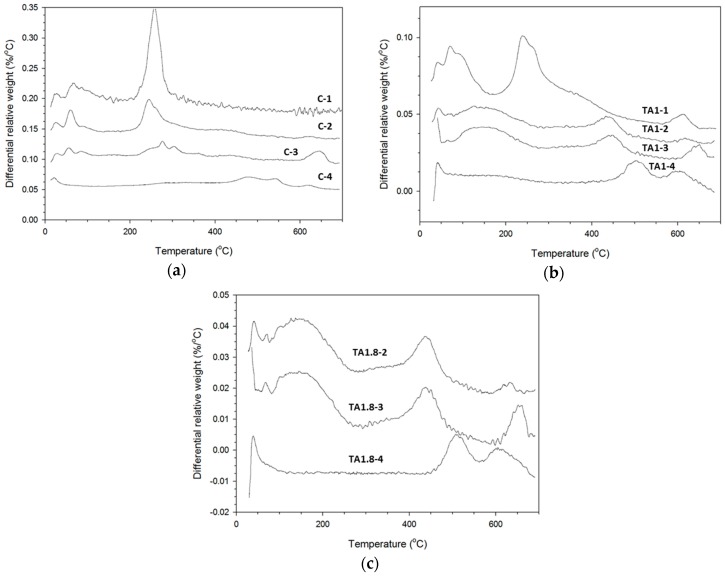
Differential thermal gravimetric analyses (DTG) of TSRC control (**a**) specimens and specimens with (**b**) 1%; and (**c**) 1.8% tartaric acid.

TGA analyses were performed to complement the results of phase composition determined by the XRD method. Figure 8a compares the TGA curves of the control specimen cured under different conditions. The peaks below about 150 °C can be attributed to poorly crystallized (sodium, calcium)-aluminosilicate hydrates [28,45,46] along with the water loss from zeolite A [47] and hydroxysodalite [48]. These peaks are sharper for 200 °C-treated cement (C-2), suggesting a higher crystallinity of these phases. They disappear after the curing at 300 °C (C-4). The peaks between 230 and 260 °C are from gibbsite [49,50] with the peaks’ intensity and temperature decreasing as the curing temperature and time increase to 200 °C and 7 days (C-2,3). In agreement with the XRD results, this peak is lacking for the specimen cured at 300 °C (C-4). The decomposition of members of the hydrogrossular family follows the gibbsite peak [49,51,52]; katoite gives right shoulders on the gibbsite peak (C-1,2) and becomes a separate peak at 280 °C after a 7-d curing at 200 °C. No weight loss is associated with this temperature for the 300 °C-cured sample (C-4). Further weight-loss peaks can be related to boehmite (307 to 442 °C) [53], with a possible contribution from gismondine [44]. The sample cured at 300 °C does not lose any weight until about 479 °C when a wide peak is followed by another peak at 543 °C. These peaks are attributable to analcimes that may lose weight in a wide range of high temperatures from 200 to 700 °C with the increased CaO content shifting the dehydration temperature to higher values [54] and/or to boehmite that loses the hydroxyl groups between 400 and 550 °C [53,55].

In agreement with the XRD data, the TGA curves of the retarded specimens had either a much smaller gibbsite decomposition peak at 237 °C (Figure 8b, TA1-1) or did not have any gibbsite peaks (all other retarded samples). Boehmite dehydroxylation peaks appeared around 440 °C for the 1% TA sample and at 434 °C for 1.8% TA samples cured at 200 °C (Figure 7). These peaks shifted to above 500 °C after curing at 300 °C, indicating larger crystals or/and higher crystallinity. A big, wide dehydration peak around 135 °C for 1% TA samples and at 140 °C for 1.8% TA samples can be ascribed to the dehydration of (N, C)–A–S–H and water loss from sodalites. There was no peak associated with water bound in calcium aluminosilicates for samples cured at 200 and 300 °C. Peaks between 600 and 700 °C were attributed to carbonates with possible contribution from analcime series for the 300 °C-cured specimen [54].

Because of the blends’ complexity, decomposition peaks overlap in the TGA, and single-phase identification is problematic. However, TGA tests confirmed suppression of transitional, low-temperature phases in retarded specimens, showing a much lower gibbsite content after curing for 22 days at 85 °C and 1 day at 200 °C in comparison with the control specimens cured under the same conditions.

### 3.4. Microstructural Characterization

**Figure 9:**
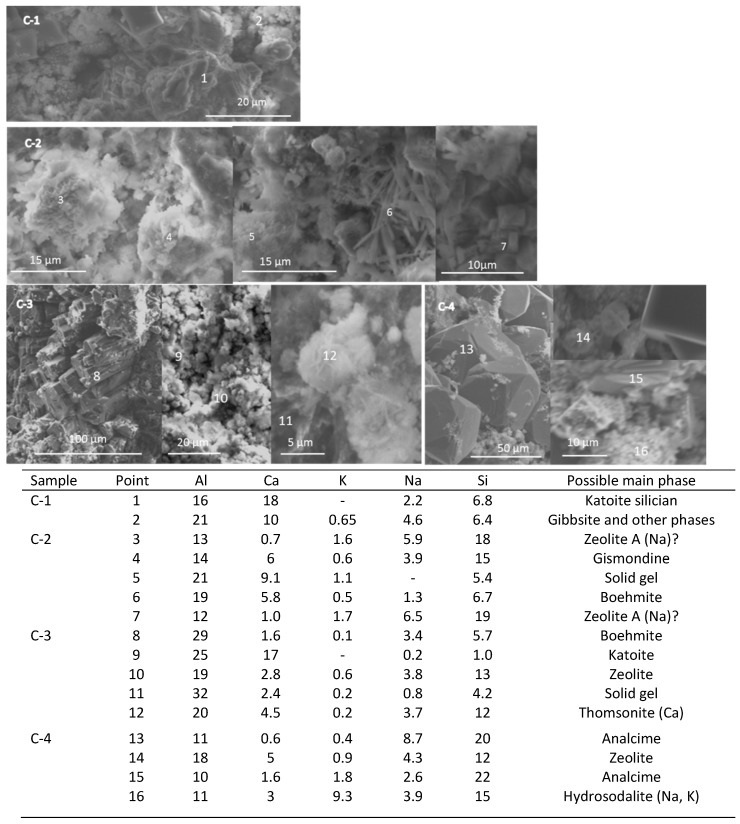
Scanning electron micrographs and point compositions of typical cement microstructures for control TSRC specimens without tartaric acid.

The SEM-EDX studies on freshly fractured samples were conducted to support the XRD findings and to observe microstructures of cement specimens cured under different conditions with and without the retarder. Although cement is highly inhomogeneous and signals from multiple phases overlap, a combination of morphological features and atomic composition allowed identification of specific phases in some cases. Figure 9 shows scanning electron micrographs of a control sample cured at different temperatures and corresponding composition in atomic percent at individual test points. The sample cured for 22 days at 85 °C (C-1) revealed a group of randomly precipitated individual crystals intermixed with poorly crystallized phases, partially reacted fly-ash particles covered with the reaction products, and an Al-rich hydrate. In fact, there was a strong aluminum signal at all tested locations, which might be due to the massive and fast precipitation of gibbsite. This complicated identification of other crystalline phases in most of the locations. The atomic composition of big crystalline clusters corresponded to Si-substituted katoite (point 1), and the well-formed cubic crystals were likely zeolite A.

The micrograph of the sample cured for 3 days at 85 °C and 1 day at 200 °C (C-2) showed groups of crystals with atomic composition close to zeolite A (Na) (points 3 and 7); a cluster of crystals identified as gismondine, based on their atomic content (point 4); aluminum-rich solid-gel structure; and needle-like aluminum-rich crystals, which probably were boehmite, in agreement with the XRD results for this specimen (point 6).

A longer curing time of 7 days at 200 °C (C-3) generated large boehmite crystals (point 8) among the smaller katoite (point 9) and zeolite phases (points 10). In agreement with the XRD results, the atomic composition suggested the presence of thomsonite (Ca) in the specimen (point 12). The solid-gel hydrate was rich in aluminum (point 11). 

Large analcime crystals dominated the morphology of the control specimen cured at 300 °C (C-4) [56,57,58]. These crystals varied from big cubo-octahedral ones to cubic symmetries (points 13 and 15). Small crystals, including those with atomic composition of K, Na-hydrosodalite, were spread among the analcime (point 16).

**Figure 10:**
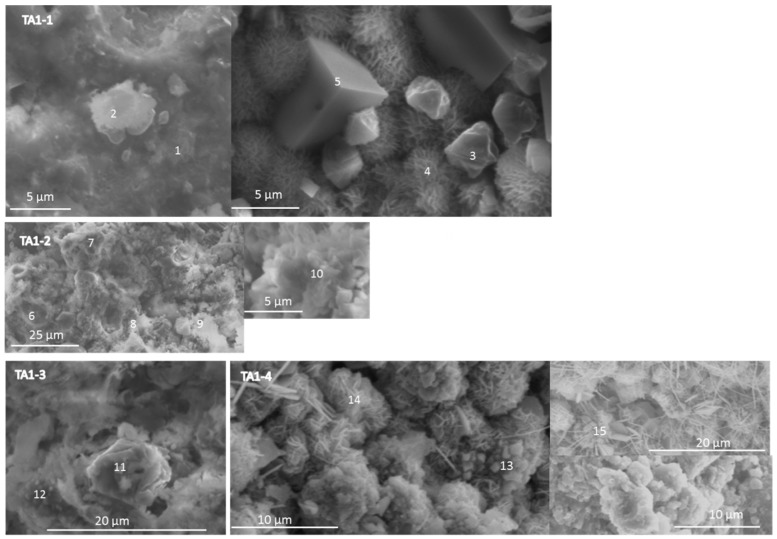

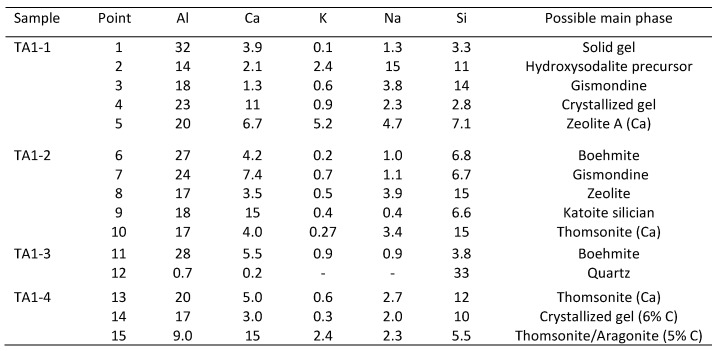
Scanning electron micrographs and point compositions of typical cement microstructures for TSRC specimens with 1% by weight of blend TA.

Figure 10 shows scanning electron micrographs of the specimen retarded with 1% TA. After 22 days at 85 °C (TA1-1), most of the freshly broken surface of this specimen looked like a solidified liquid with some crystals precipitated in it. This solidified noncrystalline matrix was very rich in aluminum (point 1). Some brighter particles had a typical composition of hydroxysodalite (point 2). Elemental-composition analyses of these small particles were problematic because of their mobility under the high-energy EDX beam suggesting a sodium-rich composition. Larger cubic crystals with atomic composition of zeolite A (point 5) and smaller crystals, possibly of gismondine (point 3), were surrounded by particles with rosette-like morphology and higher aluminum content (point 4).

The morphology of the specimen cured for a day at 200 °C (TA1-2) was a dense matrix with inclusions of small crystals with compositions typical for boehmite (point 6), gismondine (point 7), and katoite with silicon (point 9). A small cluster of poorly crystallized particles had a typical thomsonite (Ca) atomic composition (point 10).

After 7 days at 200 °C (TA1-3), the specimen still had a dense matrix with bigger clusters of aluminum-rich particles, possibly boehmite precursors (11). The quartz-rich small particles appeared in the sample (12), along with amorphous spots of typical thomsonite (Ca) composition.

The sample treated at 300 °C (TA1-4) was composed of thomsonite-like crystals (points 13) covered with fibrous material, likely aragonite, in some locations (point 15) in a more or less crystalized matrix rich in aluminum and silicon (point 14). 

**Figure 11:**
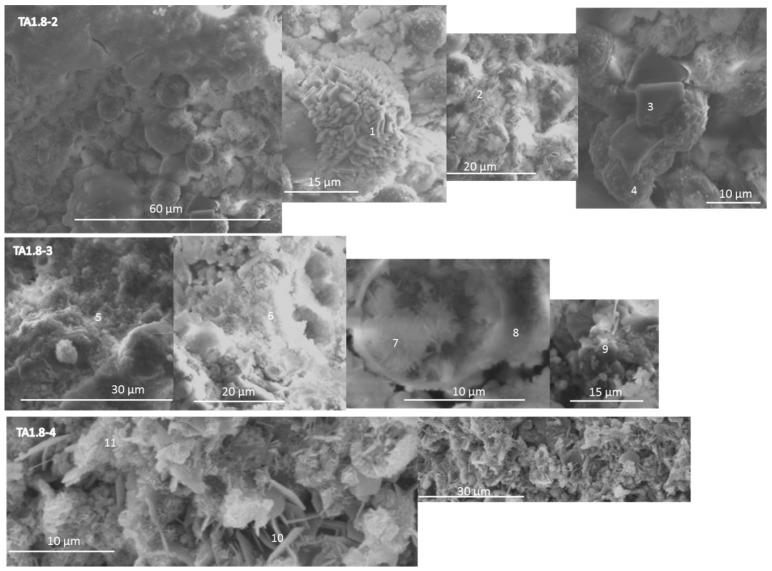

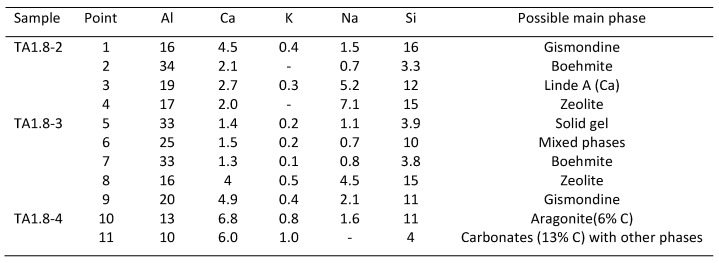
Scanning electron micrographs and point compositions of typical cement microstructures for TSRC specimens with 1.8% by weight of blend TA.

Figure 11 of the 1.8% TA specimen cured at 200 °C for 1 day (TA1.8-2) indicates a solid-gel matrix covering nonreacted or partially reacted fly ash particles intermixed with the crystalline reaction products. The atomic composition and morphology of crystals grown on the fly ash was typical of gismondine (point 1). The needle-like aluminum-rich crystals randomly coating the fly ash probably were boehmite (point 2). Cubic crystals, likely of zeolite A (Ca) (point 3) had some other zeolite with higher sodium and silicon content growing on their surface (point 4).

Longer curing times at 200 °C (TA1.8-3) did not bring about major changes in the sample’s morphology, which was a solid gel with inclusions of some small crystals (point 5). The poorly crystalized matrix was rich in both aluminum and silicon (point 6). There were boehmite crystals growing in the available space inside partially reacted fly ash particles (point 7), while the outer shell of fly ash was covered with the silicon and sodium-rich zeolite-precursor (point 8). Small clusters with gismondine-like atomic composition also were detected (point 9).

The freshly-broken surface of the 1.8% TA sample cured at 300 °C (TA1.8-4) was covered with plate-like carbon-rich phases, and the underlying structures were difficult to observe and analyze.

There is a general agreement between the SEM/EDX and XRD results. The former confirmed the presence of gismondine in retarded specimens and the formation of zeolites and Al-rich phases. The measurements of atomic composition suggest the presence of calcium in the structures of zeolite A and thomsonite and the partial substitution of hydroxyl groups in katoite with silicate. Large boehmite crystals were observed in the control specimen; they were surrounded by randomly precipitated crystals at the curing temperature of 200 °C. Smaller crystals precipitated among nonreacted fly ash particles and grew inside them at longer curing times/higher temperature in the presence of tartaric acid under similar conditions. Solid-gel-type morphology rather than that of precipitated crystals distinguished the retarded specimens. This solid gel was very aluminum rich.

## 4. Discussion

Retarders may affect kinetics of a cement’s dissolution, product formation, growth, and crystallization, changing the pore-water speciation, pores structure and the products formed.

For TSRC retarded with tartaric acid, the speciation changes may conceivably take place due to the formation of metal-organic complexes that, for the time of their existence, remove the metals from the pool of ions available for building the crystals [23]. Metal-organic interactions may cause slow dissolution, nucleation, and/or growth of hydrates, resulting in their delayed precipitation. In the presence of tartaric acid, the pore-water concentration of calcium after the first 2 h of hydration was almost 10 times higher than for the control sample, in which both calcium and aluminum rapidly precipitated on the surface of the cement particles. The delayed precipitation can also explain the measured larger porosity in the retarded system. Based on the organic carbon measurements, it was estimated that 67% of the retarder was present in the pore solution along with calcium. In addition to calcium, other metals such as alkalis and aluminum may interact with the acid. Tartaric acid can complex up to five sodium atoms [59] and promote the polycondensation reaction of sodium metasilicate, which, in turn, can slow down the hydration of calcium aluminate cement and the activation of fly ash [23,24,25,26,27,28]. 

The limited availability of calcium ions in precipitated hydration products would result in preferential crystallization of phases with lower calcium content in retarded cement. XRD data support such a hypothesis. The hydrogrossular and zeolite families of hydration products include katoites that have three calcium atoms per molecule and gismondine with one calcium atom for specimens cured for 22 days at 85 °C. Comparison of their XRD peaks indicates about 30% higher peak intensity of katoites in control specimens than in 1% TA-retarded samples. This fact is not solely due to a delay in crystallization caused by tartaric acid since the peak intensity of gismondine (2θ ~ 33.2°), which has lower calcium content, is about 20% higher for the retarded sample. (The intensities of gismondine peaks were adjusted for the possible contribution of mullite based on the mullite peaks at 2θ 16.46.) Similarly, thomsonite-calcium crystallization is inhibited in the retarded specimens up to 300 °C whereas Na,K-hydroxysodalite crystalizes even after short, 1-day curing, at 200 °C. In contrast, thomsonite is one of the major crystalline phases in the control specimen after 7 days at 200 °C. Thus, the retarder enhances the crystallization of sodalite while inhibiting that of thomsonite. These observations support the hypothesis of preferential crystallization of phases with lower calcium content in the presence of tartaric acid. 

After longer hydration times, thomsonite-calcium crystallization also may occur in the presence of the retarder, but not necessarily as a major phase since it is likely to crystallize from the amorphous gel in the matrix of other high-temperature stable crystals.

The lower calcium content of the amorphous aluminosilicate and crystalline products in the presence of the retarder also could be an outcome of delayed CAC hydration that supplies calcium and additional aluminum to the gel [18]. 

To avoid mechanical stress and materials damage, it is desirable to minimize phase transitions in set cement. Fast crystallization of hydration products during the hydration of nonretarded cement happens at temperatures that may be well below the target values. The necessary phase transitions to high-temperature-stable phases follow as the temperature increases. For zeolites, it happens for the zeolite A–thomsonite transition as the temperature increases from 85 to 200 °C and to analcime for further temperature increases to 300 °C. Katoite becomes unstable at curing temperatures above 200 °C. The intensity of its peaks decreases in the XRD patterns of 7-day 200 °C-cured sample, and they vanish from the pattern of a 300 °C-cured cement. For aluminum hydroxides, gibbsite transforms to boehmite as the temperature increases. This happens in a well-crystallized matrix of the sample that undergoes a fast set without the retarder. The SEM observations showed the growth of large boehmite crystals breaking the matrix of the sample at 200 °C. All the experimental results agree that tartaric acid delays gibbsite crystallization. Even after 22 days of hydration at 85 °C, the amount of crystallized gibbsite is almost 90% higher in the control than in the specimen with 1% tartaric acid, as can be inferred from the gibbsite peak’s intensities at 2θ 18.34° normalized to the peak of corundum at 25.62°. The aluminum of the retarded samples stays amorphous for longer times (boehmite halo, low intensities of crystalline gibbsite and boehmite), and the crystallization of boehmite takes place in a largely amorphous matrix, which may more easily accommodate the new crystals without creating internal stresses. CT results showing a smaller volume of large pores (above 500 micron) in the retarded samples support these arguments. 

The CT findings suggested that the important pore range that may play a dominant role in compressive strength of the samples is above 500 microns. The porosity from these large pores was found to be inversely proportional to the compressive strength. As a result, although the use of the retarder increased the total porosity, it reduced the large pores, which also seemed to contribute to the increase in strength. Importantly, pores remained non-connected in retarded samples, which is a very desirable feature for keeping cements major function as a zonal isolation material in subterranean wells.

Cement shrinkage during the fast hydration of nonretarded cement may also cause high stress of cement matrix leading to samples cracking. The slower cement hydration allows gradual shrinkage and as a result decreased micro size cracking, which may be one of the reasons for greater compressive strength of the retarded samples notwithstanding their slightly higher porosity. 

Long-term curing would be needed to establish whether the crystallization from the amorphous matrix would proceed in the retarded specimens or it would persist in an amorphous state stabilized by the retarder. 

## 5. Conclusions

The main results of this study of an alkali-activated blend of calcium aluminate cement and fly ash F hydration with or without tartaric acid at 85, 200, and 300 °C are as follows:

1. The crystal formation and growth are delayed in the presence of tartaric acid, so the major products of the cement hydration at short curing times at 200 and 300 °C and longer curing times at 85 °C are aluminum-rich calcium aluminosilicate hydrate (C,N–A–S–H) gel along with smaller amounts of boehmite and zeolites.
2. In the control sepcimens the calcium-aluminosilicate hydrate gel is still the major product along with gibbsite at 85 °C or boehmite and zeolites as minor products at temperatures up to 200 °C; however, the cement matrix is much more crystallized, especially at 300 °C. Importantly, a number of phase transitions take place as the curing temperature rises. Boehmite, thomsonite, gismondine, and sodalite develop at 200 °C replacing gibbsite, zeolite A, and katoite that form at 85 °C. Analcime becomes the major crystalline phase for the curing temperature of 300 °C.
3. Tartaric acid slows down the crystallization of hydration products and affects the composition of the crystalline phase. The retarder delays and/or inhibits crystallization of gibbsite, katoite, and thomsonite, while favoring formation of high-temperature-stable, low-calcium zeolite, sodalite, and hydroxyaluminum oxide, boehmite, limiting phase transitions with the temperature increase.
4. Despite the increase in the total porosity the mechanical properties of the retarded cement are comparable to the control after 200 °C curing and improved after 300 °C. The porosity increase is goverened by the pores of less than 500 micron while the volume of the larger pores actually decreases when the retarder is used.

## Figures and Tables

**Table 1:** The elemental composition of Thermal Shock Resistant Cement components.

Component	Calcium Aluminate Cement	Flay Ash Class F	Sodium Metasilicate
Al_2_O_3_	73.8	34.8	-
CaO	26.1	2.7	-
SiO_2_	-	50.4	46.6
Fe_2_O_3_	0.1	7.1	-
Na_2_O	-	0.3	50.5
K_2_O	-	3.1	-
TiO_2_	-	1.6	-

**Table 2:** Specimens’ curing conditions.

Specimen	Curing Conditions			
	22 Days 85 °C Atm. Pressure	3 Days 85 °C, 1 Day 200 °C 6.89 MPa	3 Days 85 °C, 7 Days 200 °C 6.89 MPa	3 Days 85 °C, 1 Day 300 °C 8.27 MPa
TSRC without retarder (control)	C-1	C-2	C-3	C-4
TSRC with 1% ^1^ TA	TA1-1	TA1-2	TA1-3	TA1-4
TSRC with 1.8% ^1^ TA	-	TA1.8-2	TA1.8-3	TA1.8-4

^1^ by weight of blend.

**Table 3:** Compressive strength, toughness, and porosity of control- and retarded- TSRC specimens.

Specimen	Compressive Strength (MPa)	Toughness (N-mm/mm^3^)	Porosity (%)
**3 days 85 °C, 1 day 200 °C**			
C-2	9.9 ± 0.8	0.08 ± 0.04	5.1
TA1-2	11.5 ± 2.3	0.09 ± 0.03	n.d. ^1^
TA1.8-2	10.4 ± 0.7	0.12 ± 0.05	6.1
**3 days 85 °C, 1 day 300 °C**			
C-4	4.6 ± 0.3	0.08 ± 0.04	5.4
TA1-4	7.5 ± 1.2	0.31 ± 0.19	6.1
TA1.8-4	9.4 ± 1.7	0.35 ± 0.25	n.d.

^1^ not determined.

## Abbreviations

The following abbreviations are used in this manuscript:

TA	Tartaric Acid
CAC	Calcium Aluminate Cement
FAF	Flay Ash type F
SMS	Sodium Meta-Silicate
